# Targeting Dental Pulp Stem Cell Senescence: A Potential Strategy for Improving Regenerative Endodontic Procedures

**DOI:** 10.3390/bioengineering13060654

**Published:** 2026-05-31

**Authors:** Jacob Christoffersen, Lars Bjørndal, Sally Dabelsteen

**Affiliations:** 1 Oral Pathology and Medicine, Section for Oral Biology and Immunopathology and Center for Glycocalyx Research, Department of Odontology, University of Copenhagen, DK-2200 Copenhagen, Denmark; kzg351@alumni.ku.dk; 2 Cariology and Endodontics, Section for Clinical Oral Microbiology, Department of Odontology, University of Copenhagen, DK-2200 Copenhagen, Denmark; labj@sund.ku.dk

**Keywords:** senescence, cellular aging, dental pulp stem cells, regenerative dentistry

## Abstract

The effects of aging on dental pulp stem cells (DPSCs) have been extensively investigated in recent years. Senescent DPSCs may negatively affect pulpal regeneration in the aged tooth and be a driver of chronic inflammation. In the near future, targeting key inducers of cellular senescence may therefore become central to the development of new strategies to enhance the regenerative capacities of DPSCs. The aim of this perspective article is to provide an overview of the molecular mechanisms driving cellular senescence in DPSCs. Furthermore, potential clinical applications of targeting DPSC senescence in regenerative endodontics are discussed, including strategies to preserve exposed pulp, promote pulp-like tissue through revascularization or revitalization, and achieve complete pulp regeneration.

## 1. Introduction

**Figure 1:**
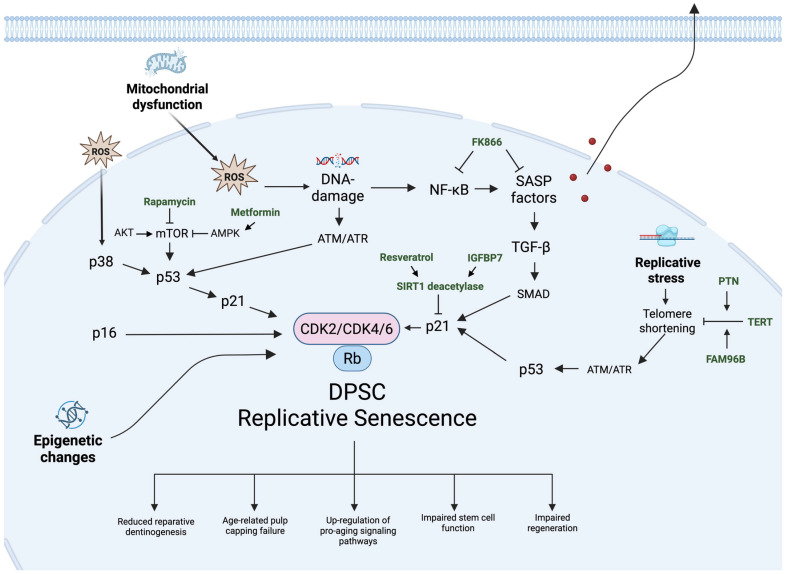
Induction and regulation of signaling pathways leading to cellular DPSC senescence. Anti-senescent regulatory mechanisms are highlighted in green in Figure 1 with inhibitory interactions indicated by inhibitory arrows. Created in BioRender. Christoffersen, J. (2026).

Dental pulp stem cells (DPSCs) are located in the perivascular niche of the dental pulp and represent a postnatal population with mesenchymal stem cell (MSC)-like properties [1,2]. In a young stem cell niche environment, the balance between self-renewal and differentiation of stem cells is fine-tuned, and staying in a quiescent mode enables them to stay ready for regenerating tissue in response to stressful events [3]. However, cellular aging can alter this reversible control mechanism to become an irreversible state of growth arrest, leading to reduced regeneration and tissue repair. Cellular senescence describes such a stable and terminal state of growth arrest in which cells lose their proliferative capacity [4]. A major concern regarding the use of DPSCs in mesenchymal stem cell (MSC)–based therapies is, however, the senescence-associated decline in proliferative capacity, which can become a challenge for in vitro expansion before implantation [5]. In addition, any accumulation of senescent DPSCs may compromise cell-free regenerative endodontic procedures and hinder efforts to improve existing vital pulp therapies. In future endodontics, targeting cellular senescence pathways may therefore contribute to preserving exposed dental pulp and restoring the regenerative capacity of older teeth to that of young, healthy teeth. Furthermore, targeting DPSC senescence may facilitate re-establishment of vital tissue in a previously necrotic pulp space, thereby promoting pulp-like tissue formation—commonly described as revascularization or revitalization—or even regenerating the complete pulp. The aim of this work is to integrate recent advances in DPSC senescence research with emerging clinical approaches in vital pulp therapy and regenerative endodontic procedures, thereby highlighting implications for future endodontic practice. Section 4 provides an overview of key pathways regulating DPSC senescence and their underlying mechanisms, encompassing pro-senescent, anti-senescent, and context-dependent dual effects. While some pathways promote senescence, others act as counter-regulatory mechanisms with relevance for improving future endodontic therapies. These anti-senescent regulatory mechanisms are highlighted in green in Figure 1 with inhibitory interactions indicated by inhibitory arrows. Section 5 explores potential therapeutic strategies aimed at enhancing regenerative and repair processes in the aged tooth. Given the substantial heterogeneity in experimental models, methods, intervention strategies, and outcome measures across the current literature, the present article is structured as a perspective article rather than a systematic review.

## 2. Literature Search Strategy

A literature search was conducted to identify relevant original studies on DPSC senescence. No formal systematic review methodology was applied. Inclusion criteria comprised original studies investigating senescence mechanisms in dental pulp stem cells (DPSCs), including both in vivo and in vitro findings, as well as studies using the non-specific term “dental pulp cells” (DPCs) when DPSCs were clearly specified. Exclusion criteria included studies primarily focused on age estimation methods and studies investigating the clinical application of DPSCs in non-odontological medical fields. The clinical aspects of DPSC senescence discussed in the final section (Section 5) are based on studies published in recent years.

## 3. Inducers of Cellular Senescence

Distinguishing between the causes and consequences of cellular senescence can be challenging; however, it is well established that prominent inducers of senescence include mitochondrial dysfunction, which leads to the accumulation of reactive oxygen species (ROS) and DNA damage [6,7], replicative stress caused by telomere shortening [8], oncogene activation [9], and epigenetic modifications [10]. Traditionally, cellular senescence was mainly seen as a cancer-preventing cell-cycle arrest; however, in recent years, increasing research attention has focused on its impact on tissue homeostasis and regeneration [10]. Results from in vitro studies have revealed significant signs of cellular senescence in DPSCs, including senescence-associated β-galactosidase and the expression of proteins such as p21 and p53 [11]. However, physiological oxygen levels (pO_2_ 3–6%) induce less premature senescence than atmospheric oxygen levels (pO_2_ 21%) [12], suggesting that the stem cell niche may protect the dental pulp by reducing ROS levels and activating HIF transcription factors [13]. Nevertheless, senescence-inducing stressors can disrupt the niche through SASP, while elimination of senescent cells may restore stem cell function and rejuvenate the microenvironment [14].

## 4. Pathways Controlling DPSC Senescence

### 4.1. The p53-p21 Pathway

The processes of cellular senescence in DPSCs are modulated by highly specific regulatory pathways that promote or suppress its downstream effects. The p53-p21 pathway is central in regulating whether the cell commits to cell division or not. During the cell cycle, p53 prevents unchecked cell proliferation by binding to a regulatory region of the p21 gene, which inhibits Cdks and arrests the cell cycle [4]. This acts as a fine-tuned cellular control system, whereas prolonged activation can be harmful and lead to tissue aging, impaired regeneration, and chronic inflammation [3].

#### 4.1.1. Anti-Aging Role of Sirtuins

In recent years, the sirtuin (SIRT) protein family has gained increased attention for its anti-aging effects. To date, seven members of the sirtuin family have been identified [15], two of which are implicated in modulating DPSC senescence by affecting p21 transcription. SIRT1 is described as the most upregulated deacetylase in response to overexpression of insulin-like growth factor binding proteins-7 (IGFBP7), and has been shown to induce deacetylation of H3K36ac, reducing the binding affinity of H3K36ac for the p21 promoter region, thereby decreasing p21 transcription [16]. On the other hand, SIRT7 has been implicated in DPSC senescence, with one study reporting that SIRT7-mediated desuccinylation of ROCK1 induces senescence [17], and another study showing that inhibition of SIRT7 expression by miR-152 suppresses cellular senescence in DPSCs [18].

#### 4.1.2. mTOR Signaling

The mechanistic target of rapamycin (mTOR) signaling pathway can activate the p53–p21 pathway, while rapamycin has been shown to act as an mTOR inhibitor [19]. Metformin is thought to activate AMPK, resulting in mTOR inhibition through targeting of CAB39 [20]. Although p53 acts as a canonical tumor suppressor that inhibits the cell cycle, in certain contexts, loss of a tumor suppressor gene may also reduce cell proliferation through activation of the mTOR signaling pathway. Studies have demonstrated that loss of the tumor suppressor gene PTEN induces senescence in mouse models and in human tumor xenografts through effects on the mTOR–p53 signaling pathway [21]. PTEN is a major negative regulator of the PI3K/Akt/mTOR pathway. Loss of PTEN causes over-activation of mTOR, leading to phosphorylation of p53 and increased p53 stability, thereby inducing senescence via p21 accumulation [22]. Studies in normal human fibroblasts show that mTORC1 specifically is required for AKT-induced senescence, and thus inhibiting mTOR can disrupt the senescence brake on cancer-initiating cells [23]. However, in the context of tissue rejuvenation, mTOR inhibition by rapamycin can be beneficial for regeneration. In DPSCs, it has recently been shown that mTOR inhibition through Akt, a downstream target of ILK involved in mTOR regulation, can be achieved by direct inhibition of ILK [19]. Therefore, the regulation of cell division with respect to tumor suppressors is complex, and the coordination of these pathways is highly relevant for the capacity of DPSCs to undergo self-renewal and proliferation.

#### 4.1.3. Telomere Shortening

Another regulator of the p53 protein is telomere shortening. Telomeres are vital for genomic stability, and their shortening leads to permanent proliferation arrest, whereas telomere length regulation via telomerase activity inhibits the process of replicative senescence [24]. Replicative stress and shortening of telomeres leading to activation of the p53-p21 pathway are inhibited by telomerase reverse transcriptase (TERT), which encodes the telomerase enzyme, but in addition to deactivating p53, TERT also alters p16 expression [25]. In DPSCs, key regulators of TERT have been shown to be FAM96B and PTN [26,27]. Interestingly, stem cells from the apical papilla (SCAP) exhibit higher telomerase activity than DPSCs, indicating a higher proliferative capacity of this cell type [28].

### 4.2. The p38 MAPK Pathway

As part of the p38 MAPK pathway, the p16 protein is involved in regulating cellular senescence, and it has been shown that activation of p38 MAPK by elevated levels of reactive oxygen species (ROS) can shorten the lifespan of hematopoietic stem cells [29]. Other studies have demonstrated that p16 protein upregulation is positively regulated by the p38 MAPK pathway, while inactivation of p38 MAPK may increase the risk of tumor formation due to uncontrolled cell proliferation [30]. However, studies in other cell types have shown that inhibition of p38 MAPK activation can also suppress invasive tumor growth [31]. In DPSCs, the regulation of senescence mechanisms via p38 MAPK is complex, as phosphorylation of p38 is not necessarily associated with senescence but has also been reported in connection with inhibition of senescence in DPSCs [27]. Although activation of p38 MAPK can inhibit proliferation via the p16 protein, exosome-mediated activation of the MAPK pathway in DPSCs has also been shown to increase proliferation [32]. This dual effect may mechanistically be explained by the degree of p38 activation, as low levels of p38 activation promote cell survival, whereas sustained and intense p38 activation leads to senescence [33]. Studies have shown that recombinant pleiotrophin (PTN) can attenuate the consequences of senescence in DPSCs by modulating the p38 MAPK signaling pathway [27]. Mechanistically, PTN silencing downregulates phosphorylated-p38 MAPK (p-p38 MAPK) expression without altering total p38 MAPK levels, whereas addition of recombinant PTN protein restores p-p38 MAPK levels in PTN-silenced DPSCs [27].

### 4.3. SASP Factors

The mechanisms of DPSC senescence described above primarily involve intracellular pathways. However, DPSC senescence can also be propagated by communication between senescent and neighboring cells. In DPSCs, H_2_O_2_-induced premature senescence has been associated with activation of the nuclear factor-kappa B (NF-κB), which regulates the transcription of NF-κB-dependent senescence-associated secretory phenotype (SASP) genes [11]. SASP factors may contribute to tissue repair and regeneration if they are rapidly cleared by immune cells. In contrast, long-term exposure to SASP factors can lead to age-related diseases and chronic inflammation in the stem cell niche [34]. In DPSCs, ROS-induced NF-κB activation may therefore contribute to a pro-inflammatory microenvironment by promoting the secretion of SASP factors that influence neighboring DPSCs. Within the stem cell niche, such paracrine signaling may induce niche deterioration and further drive the progression of senescence [3]. A study in fibroblasts has shown that a natural plant-derived polyphenol such as resveratrol can exert anti-SASP activity [35]. Although resveratrol has been reported to delay senescence in DPSCs through SIRT-1 activation, the authors did not specifically analyze SASP as an underlying mechanism of its action in DPSCs [36]. In contrast, treatment of DPSCs with FK866 has been shown to reverse NF-κB-activation and reduce SASP factor expression [11].

### 4.4. Epigenetics

Studying SASP activity in DPSCs may also provide insight into the epigenetic regulation of DPSC senescence, including the epigenetic regulation of SASP-associated genes. Epigenetics is considered a key hallmark of cellular aging and is mediated, in part, by DNA methylation and histone modifications [37]. In late-passage DPSCs, a higher frequency of hypomethylated Alu CpG sites has been observed compared with early-passage DPSCs. Since DNA methylation generally represses gene expression, this hypomethylation has been associated with age-related inhibition of DPSC proliferation [38]. In addition, serine metabolism has been linked to upregulation of the senescence marker p16 via DNA hypomethylation [39].

## 5. Therapeutic Strategies Targeting DPSC Senescence

The differentiation potential of DPSCs declines with age, affecting odontogenic, osteogenic, neurogenic, and adipogenic lineages [40]. Furthermore, cellular senescence also impairs DPSC migration, angiogenic signaling, and differentiation, ultimately resulting in reduced regenerative capacities of DPSCs. Age-related changes in stem cell functions of DPSCs may compromise not only the tooth’s repair mechanisms but also hinder the development of future regenerative endodontic procedures. Therefore, considering the distinct biological contexts of repair and regeneration, the impact of DPSC senescence should be evaluated within the two different approaches: vital pulp therapy and regenerative endodontic procedures (REP).

### 5.1. Targeting DPSC Senescence in Vital Pulp Therapies

The dentine–pulp complex forms a unique biological unit possessing defensive and reparative responses to tooth irritation [41]. In cases of carious pulp exposure, the prognosis for repair is often unfavorable, challenging the rationale for direct pulp capping in asymptomatic adult posterior teeth with carious pulp exposure [42]. Although short-term outcomes may appear promising, long-term prognosis is poor; after 4 years, success rates remain low even when *Lege Artis* pulp capping protocols are followed [42]. These findings are supported by Bjørndal et al. 2017, who reported successful outcomes in only 9% of patients undergoing conventional pulp capping after pulp exposure in deep carious lesions [43].

Despite the uncertain prognosis of pulp capping, the tooth may still attempt to form a protective dentinal bridge following pulp exposure, a process that depends on the recruitment and differentiation of DPSCs from their niche. Interestingly, the quality of the mineralized dentinal bridge formed by mineral-secreting cells varies not only according to the pulp capping material used but also with patient age [44,45,46,47]. In one study, patients older than 40 years had six times higher odds of pulp capping failure than patients younger than 40 years [45]. However, no pulp capping material has yet been specifically designed to target age-related impairment of DPSC reparative capacity.

In 2024, Lee et al. studied specific age-related mechanisms in mice and, using Cpne7- knockout models, demonstrated that Cpne7 deletion causes premature aging of the pulpal microenvironment. They further showed that intraperitoneal injection of the CPNE7-derived functional peptide rescued this premature aging phenotype [48]. According to the authors, CPNE7-DP restores compromised cell survival by promoting DPSC proliferation and odontogenic differentiation [48]. In addition, a recent study has demonstrated that the silicate-containing dental material Biodentine™ may exert some anti-aging effects on human DPSCs through the Wnt/β-catenin pathway by downregulating p21, p53 and p16 [49]. Together, these studies indicate substantial progress in identifying both novel and established strategies to preserve tooth vitality by targeting cellular senescence.

Efforts to improve the reparative potential of DPSCs in vital pulp therapies raise an important question: When can we consider the dental pulp biologically capable of healing and forming reparative dentine? In recent years, various predictors of DPSC aging have been identified in vitro. These aging-associated markers include reduced glycolytic capacity and impaired lipid utilization as an energy source in rapid aging DPSCs, which correlate with upregulation of the TGF-beta pathway [50]. Since DPSCs derived from donors of the same chronological age may exhibit variable aging rates [50], predicting temporal changes in an individual patient’s stem cell population may become central in the development of future vital and regenerative pulp therapies. See Table 1 for the functional consequences of senescence in dental pulp repair and vital pulp therapy.

### 5.2. De Novo Pulp Regeneration

To regenerate tissue within a necrotic pulp space, pulp regenerative procedures (REP) have gained increasing attention and can be categorized into two main strategies: an exogenous approach using cell transplantation and a “cell-homing” approach based on endogenous cell migration [51]. Furthermore, revascularization represents a promising pulp-preserving regenerative strategy. At present, only pulp-like tissue is observed without an actual odontoblastic region; however, in theory, complete pulp regeneration may be achieved by stimulating epithelial–mesenchymal interactions analogous to those occurring during tooth formation to ensure cell migration, proliferation, odontogenic differentiation, and angiogenesis [51]. Until now, REP studies have primarily focused on single-rooted, immature young teeth, but with promising outcomes [52,53,54]. This is consistent with a 2022 expert consensus stating that the clinical relevance for REPs is primarily documented in young patients with immature teeth [55]. Further research is therefore needed to determine whether comparable outcomes can be achieved in older patients.

#### 5.2.1. Cell-Free Methods

Today, the cell-homing strategy is generally considered the most clinically feasible approach for pulp regeneration, as it avoids the need for stem cell isolation, ex vivo expansion, or exogenous transplantation of stem cells [51]. In 2023, J. Shi et al. demonstrated that mesenchymal stem cell (MSC)-derived exosomes enhance dental pulp stem cell proliferation, suggesting a potential cell-free regenerative approach to counteract senescence [56]. According to the authors, MSC-derived exosomes may restore pulp homeostasis and promote pulp-dentin regeneration [56]. Revascularization is another endodontic approach that does not require the addition of external cellular material, relying instead on induced bleeding or the use of platelet-rich plasma, platelet-rich fibrin, or platelet pellet as scaffolds [53]. Although the current literature does not provide sufficient evidence to conclude that advanced patient age is a limiting factor for REP, research has demonstrated significant differences in the angiogenic capacity of pulpal stem cells depending on their age. For example, higher levels of VEGF have been detected in dental pulp stem cells from primary teeth (SHED) than in DPSCs derived from permanent teeth [57]. Boyle et al. demonstrated that TNF-α, together with VEGF, increases DPSC proliferation while simultaneously reducing telomere length [58]. However, the growth factors present within the blood clot formed after intentional induction of apical bleeding are not sufficient to facilitate the differentiation of either stem cells from the apical papilla (SCAPs) or cells from the periradicular tissues into any component of a new, functional pulp-dentine complex [58]. Therefore, particularly in older patients with reduced vascularization and impaired regenerative potential, autologous platelet concentrates may provide a more favorable scaffold for cell differentiation and growth.

#### 5.2.2. Cell-Based Methods

To date, animal studies have shown promising results for stem cell–based pulp regeneration. In 2013, researchers demonstrated the formation of pulp-like tissue and peritubular dentine after human dental stem cells were injected into extracted roots and subsequently implanted into mice [59]. In 2017, a clinical trial documented regeneration of functional pulp tissue in traumatized teeth following autologous stem cell transplantation using cells harvested from the patient’s extracted primary canine [60]. However, clinical translation of cell-based therapies remains limited by high costs and practical implementation challenges [56] and replicative aging, which can result from long-term in vitro culture of DPSCs under ambient oxygen tension [12,61]. Interestingly, a recent study [61] using SCAPs highlighted the potential of three-dimensional (3D) culture systems to overcome limitations associated with conventional two-dimensional (2D) culture. Following transplantation of SCAPs under either 2D or 3D conditions, 3D culture delayed cellular aging, thereby addressing one of the major challenges associated with the application of this stem cell type in tissue regeneration [61]. Recently, it has also been demonstrated that pretreatment of DPSCs with nicotinamide riboside (NR), which delays senescence, significantly enhances odontoblastic differentiation in vitro and in vivo. Transplanting NR-treated DPSCs into root canals may therefore promote pulp–dentine complex regeneration by targeting cellular senescence and enhancing stem cell differentiation [62]. See Table 1 for the functional consequences of senescence in dental pulp regeneration and REP.

### 5.3. Taking the Microenvironment into Account and Future Perspectives

The identification of mechanisms underlying DPSC senescence has primarily relied on results from in vitro studies of replicative senescence. However, cells isolated from an aged donor have already been exposed to inflammaging and oxidative stress, highlighting the need for in vivo models that capture senescence-associated changes within the native stem cell microenvironment. This is supported by evidence that microenvironmental rejuvenation, rather than targeting stem cells alone, can significantly improve regenerative outcomes. As an example, trypsin treatment of pulpectomized teeth before cell transplantation has been shown to enhance pulp regeneration in aged teeth. This effect was associated with increased expression of genes involved in immunomodulation, cell survival, and ECM degradation in aged periodontal ligament cells (PDLCs), as well as indirect modification of the microenvironment through increased fibronectin release from cementum, promoting angiogenesis and migration in aged DPSCs [63]. Similarly, studies of PDLC senescence have shown that in vivo targeting of inflammatory CCR3/CCL11 signaling can enhance rejuvenation and stimulate pulp regeneration in aged dog teeth [64]. Together, these findings indicate that the age-related decline in regenerative capacity should be considered in relation to changes in the surrounding microenvironment. Targeting both the microenvironment and resident stem cells could provide new opportunities to enhance pulp regeneration in aged teeth [65]. However, future studies should carefully define regeneration and clearly specify the clinical outcome targeted when addressing cellular senescence. Accordingly, this work has discussed recent research on DPSC senescence within distinct biological contexts of repair and regeneration, emphasizing that the impact of DPSC senescence should be evaluated separately in relation to vital pulp therapy and de novo pulp regeneration.

## 6. Conclusions

DPSCs progressively lose their capacity for self-renewal, proliferation, and differentiation as a consequence of cellular senescence. These age-related changes can be driven by replicative stress, mitochondrial dysfunction, alterations in tumor suppressor genes, and epigenetic modifications. Key signaling pathways that regulate DPSC fate and lineage commitment can be modulated in vitro, and such interventions have been shown to enhance the regenerative potential of DPSCs. Consequently, age-related alterations in DPSC stem cell properties represent a critical consideration for the development of future regenerative endodontic procedures, including both cell-based and cell-free approaches. Furthermore, targeting cellular senescence of the aged tooth could potentially improve existing vital pulp therapies.

## Figures and Tables

**Table 1:** Functional consequences of senescence in dental pulp repair and regeneration. The table summarizes key points discussed in Section 4.1 and Section 4.2.

	Vital Pulp Therapy	Regenerative Endodontic Procedures (REP)
Overall functional outcome	Reduced tertiary dentin formation and poor-quality dentin bridge. High age-dependent failure rates in pulp exposures treated with pulp capping procedures.	Reduced regenerative capacity of DPSCs and impaired reconstruction of a functional pulp-dentin complex.
DPSC function	Reduced proliferation limits reparative dentinogenesis. Heterogenous aging rates among DPSCs from same age individuals. Age-related decline in reparative capacity of DPSCs.	Replicative senescence during in vitro expansion impairs stem cell function and regenerative capacity. Angiogenic capacity varies between dental pulp stem cell sources (SHED/DPSC/SCAP).
Metabolism and microenvironment	Reduced glycolytic capacity and lipid utilization in aging DPSCs, associated with impaired reparative function. Upregulation of pro-aging signaling pathways (e.g., TGF-β) linked to diminished tissue repair capacity.	Hypoxic/necrotic conditions and reduced angiogenic signaling limit tissue maturation. Insufficient clot-derived growth factors may limit differentiation of pulpal stem cells from the apical papilla (SCAPs).
Therapeutic modulation and interventions	Limited anti-senescence strategies. Biomaterials like Biodentine™ may modulate aging-related pathways. CPNE7 may promote reparative dentin formation but remains experimental.	MSC-derived exosomes to enhance proliferation and promote pulp-dentin regeneration. In older patients, autologous platelet concentrates could be used to improve scaffold quality for cell differentiation and growth. 3D culture of SCAPs has shown reduced senescence and improved regenerative potential. Senescence-targeting via nicotinamide riboside (NR) improves regenerative potential of DPSCs.

## Data Availability

No new data were created or analyzed in this study. Data sharing is not applicable to this article.
